# Levodopa-Induced Dyskinesia Is Associated with Increased Thyrotropin Releasing Hormone in the Dorsal Striatum of Hemi-Parkinsonian Rats

**DOI:** 10.1371/journal.pone.0013861

**Published:** 2010-11-10

**Authors:** Ippolita Cantuti-Castelvetri, Ledia F. Hernandez, Christine E. Keller-McGandy, Lauren R. Kett, Alex Landy, Zane R. Hollingsworth, Esen Saka, Jill R. Crittenden, Eduardo A. Nillni, Anne B. Young, David G. Standaert, Ann M. Graybiel

**Affiliations:** 1 Neurology Department, MassGeneral Institute for Neurodegenerative Disease, Massachusetts General Hospital, Charlestown, Massachusetts, United States of America; 2 Department of Brain and Cognitive Sciences, McGovern Institute for Brain Research, Massachusetts Institute of Technology, Cambridge, Massachusetts, United States of America; 3 Department of Neurology, Faculty of Medicine, Hacettepe University, Ankara, Turkey; 4 Center for Neurodegeneration and Experimental Therapeutics, University of Alabama at Birmingham, Birmingham, Alabama, United States of America; 5 Division of Endocrinology, Department of Medicine, The Warren Alpert Medical School of Brown University, Rhode Island Hospital, Providence, Rhode Island, United States of America; National Institutes of Health, United States of America

## Abstract

**Background:**

Dyskinesias associated with involuntary movements and painful muscle contractions are a common and severe complication of standard levodopa (L-DOPA, L-3,4-dihydroxyphenylalanine) therapy for Parkinson's disease. Pathologic neuroplasticity leading to hyper-responsive dopamine receptor signaling in the sensorimotor striatum is thought to underlie this currently untreatable condition.

**Methodology/Principal Findings:**

Quantitative real-time polymerase chain reaction (PCR) was employed to evaluate the molecular changes associated with L-DOPA-induced dyskinesias in Parkinson's disease. With this technique, we determined that thyrotropin releasing hormone (TRH) was greatly increased in the dopamine-depleted striatum of hemi-parkinsonian rats that developed abnormal movements in response to L-DOPA therapy, relative to the levels measured in the contralateral non-dopamine-depleted striatum, and in the striatum of non-dyskinetic control rats. ProTRH immunostaining suggested that TRH peptide levels were almost absent in the dopamine-depleted striatum of control rats that did not develop dyskinesias, but in the dyskinetic rats, proTRH immunostaining was dramatically up-regulated in the striatum, particularly in the sensorimotor striatum. This up-regulation of TRH peptide affected striatal medium spiny neurons of both the direct and indirect pathways, as well as neurons in striosomes.

**Conclusions/Significance:**

TRH is not known to be a key striatal neuromodulator, but intrastriatal injection of TRH in experimental animals can induce abnormal movements, apparently through increasing dopamine release. Our finding of a dramatic and selective up-regulation of TRH expression in the sensorimotor striatum of dyskinetic rat models suggests a TRH-mediated regulatory mechanism that may underlie the pathologic neuroplasticity driving dopamine hyper-responsivity in Parkinson's disease.

## Introduction

The loss of striatal dopamine that results from degeneration of midbrain dopamine-containing neurons is responsible for much of the motor dysfunction characteristic of Parkinson's disease (PD). Symptomatic treatment of PD patients with levodopa (L-DOPA) alleviates many of these motor symptoms. However, in conjunction with the progression of the disease, long-term L-DOPA treatment leads to the development of adverse responses including debilitating L-DOPA-induced dyskinesias (LIDs) that can include choreic, hyperkinetic movements or dystonic movements [1], [2], [3]. The frequency and severity of LIDs increase with the duration of the L-DOPA treatment and with the progression of the disease [4], and there is strong evidence indicating that LIDs result from abnormal plasticity within the striatum [3], [5], [6], [7], [8].

Of the several animal models that reproduce the features of LID, among the most extensively studied is the classic hemi-parkinsonian rat model, in which one side of the striatum is depleted of dopamine by 6-hydroxydopamine (6-OHDA) before the dyskinesia-inducing treatment with L-DOPA. We used this model here to identify genes related to pathologic neuroplasticity associated with PD and LID. We found that among the largest and most significant changes that were induced by L-DOPA in the dopamine-depleted striatum of rats was an enhancement of the mRNA for preprothyrotropin releasing hormone (TRH) [9]. This result was of particular interest, given that thyrotropin-releasing hormone (TRH), in addition to its well-known function in regulating thyroid-stimulating hormone (TSH) and thyroid function, is increasingly recognized as having neuromodulatory roles [10].

Clinically, hyperthyroidism has long been associated with hyperkinetic movement disorders, and correcting the hormonal imbalance can resolve the hyperkinesia [11], [12], [13], [14]. Thyrotoxicosis has also been reported in PD patients with severe tremor [15], [16], [17], [18], and anti-thyroid treatment has been effective in controlling ‘on-off’ phenomenon and dyskinesia in thyrotoxic patients with PD [16], [19].

To determine the neural basis for these effects of TRH and thyroid hormones, we examined the effects of dopamine depletion and subsequent L-DOPA treatment on the striatal expression of *preproTRH* mRNA by quantitative PCR (qPCR) and proTRH peptide by immunohistochemistry and by radioimmunoassay (RIA) [10], [20], [21]. Our findings demonstrate that L-DOPA treatment leading to dyskinesia-like abnormal movements is associated with marked up-regulation of *preproTRH* mRNA in the dopamine-depleted striatum and with striking up-regulation of proTRH immunostaining in striatal efferent projections to the pallidum and the substantia nigra. These findings point to dysregulation of striatal TRH expression in these major output pathways of the basal ganglia as a potential central factor in the induction of LIDs.

## Results

To induce behavioral changes similar to the dyskinesias observed in PD patients [22], [23], [24], [25], [26], we employed the well-established neurotoxic rat model of PD, in which unilateral infusion of 6-OHDA into the medial forebrain bundle, inducing loss of dopamine-containing neurons in the nigrostriatal system, was followed by chronic L-DOPA treatment. We administered L-DOPA (25 mg/kg) twice daily for 21 days and evaluated the motor behavior of rats every third day for 1 min, 20 min after the morning L-DOPA treatment. Groups of control rats were given L-DOPA treatment without prior dopamine depletion, or were given the 6-OHDA infusions to deplete dopamine levels but were then treated with saline instead of L-DOPA. Because the 6-OHDA infusions (and saline control infusions) were made unilaterally, we were able also to examine the striatum contralateral to the side of dopamine-depletion as an intra-animal control.

**Figure 1:**
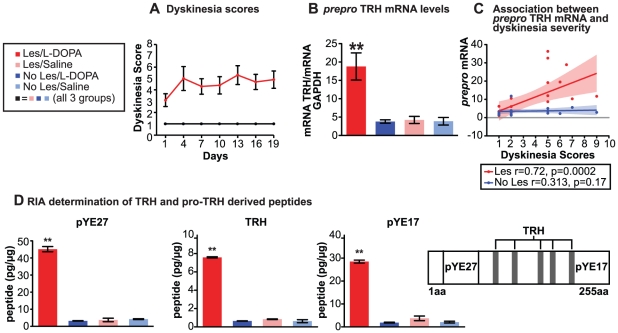
TRH increases with dyskinetic behavior. (**A**) Behavioral scores assessed 20 min after L-DOPA administration. Red line: 6-OHDA/L-DOPA. The animals belonging to this group are the only ones developing dyskinesias. Black line: superimposed, identical values for 6-OHDA/saline; No lesion/L-DOPA; no lesion/saline. The animals in the three control groups never developed dyskinesias. (6-OHDA/L-DOPA vs. all other groups p<0.0001). (**B**) *PreproTRH* mRNA levels measured by qPCR. The mRNA levels for *preproTRH* in the right striatum (6-OHDA lesion side) of the animals chronically treated with L-DOPA were significantly increased (Bonferroni T **p<0.001) with respect to the striatum contralateral to the lesion of 6-OHDA/L-DOPA rats with LIDs and the non-dyskinetic control groups. (**C**) *PreproTRH* mRNA levels are associated with the presence of dyskinesias. *PreproTRH* mRNA levels in the striatum ipsilateral to the 6-OHDA lesion showed a positive correlation with the behavioral scores (Pearson's r = 0.725, p = 0.0002), but the relationship between the behavioral scores and the mRNA levels did not follow a linear relationship. No correlation was found between the mRNA levels for *preproTRH* and the behavioral scores of the control animals (Pearson's r = 0.313, p = 0.17). Shaded areas flanking the correlation curve represent the 95% confidence intervals. (**D**) Radioimmunoassay for TRH, pYE27, and pYE17. The TRH tripeptide is derived from the processing of the larger precursor proTRH, as are the peptides pYE27, and pYE17 (TRH structure shown in panel to right). Results of the RIA show that all three peptides derived from the larger preproTRH are greatly increased in the striatum ipsilateral to the lesion in the rats with LIDs, but not in the striatum contralateral to the lesion of the rats with LIDs, or in either striata of control treatment rats (Bonferroni T p<0.001).

Of the 10 rats with unilateral 6-OHDA lesions and chronic L-DOPA treatment, 8 (80%) developed dyskinetic behaviors during the 21 days of treatment (Friedman test S  = 20.45, p<0.0001), whereas none of the animals treated with saline, or given sham lesions with or without L-DOPA treatment, developed motor complications (**Figure 1A**). The 20% of the rats with unilateral 6-OHDA infusions and chronic L-DOPA treatment that did not develop dyskinetic behaviors did not exhibit evidence of a successful nigral lesion, based on tyrosine hydroxylase (TH) immunostaining, and were excluded from further analysis.

Striatal tissues were dissected 12 hours after the last L-DOPA treatment and total RNA was extracted [27] to measure the levels of *preproTRH* mRNA with qPCR. The levels of *preproTRH* mRNA were significantly increased in the dopamine-depleted striatum of the rats that developed dyskinesia in response to the L-DOPA treatment (mixed design analysis of variance [ANOVA]; Treatment × Side interaction: F_1,31_ = 19.93, p<0.0005; **Figure 1B**), relative to levels in the contralateral striatum and to striatal levels in control rats that were dopamine-depleted and treated with saline or given sham lesions and treated with L-DOPA.

*PreproTRH* mRNA levels in the dopamine-depleted striatum of the L-DOPA-treated animals were positively correlated with the behavioral scores (Pearson's r  = 0.725, p = 0.0002) (**Figure 1C**). The *preproTRH* mRNA levels measured in the striatum contralateral to the lesion did not show a significant correlation with the behavioral scores (Pearson's r  = 0.313, p = 0.17) (**Figure 1C**). Despite the highly significant correlation between the behavioral scores and the mRNA levels in the dopamine-depleted striatum, however, the amount of *preproTRH* up-regulation and the severity of the behavioral abnormalities were not linearly related (**Figure 1C**). Thus, the up-regulation of *preproTRH* was a predictor of the presence of dyskinesia-like motor abnormalities, but not of the severity of these symptoms. These findings suggest that *preproTRH* transcription is strongly and specifically up-regulated in the dopamine-depleted striatum by L-DOPA treatment, and that this up-regulation is accompanied by the appearance of some level of abnormal movements in the animals.

### The changes in *preproTRH* mRNA are accompanied by changes in TRH peptide

To determine whether these changes in striatal *preproTRH* mRNA in the L-DOPA-treated animals were accompanied by changes in the levels of TRH peptide, we performed RIAs and examined proTRH-like immunostaining of striatal tissues from the dopamine-depleted and control sides of rats treated with L-DOPA. The biosynthesis of TRH (pyroGlu-His-ProNH2) begins with mRNA-directed ribosomal translation of a larger inactive precursor called proTRH (**Figure 1D**). ProTRH then undergoes post-translational sequence-specific cleavages in tissue- and cellular compartment-specific steps by the processing enzymes prohormone convertase 1 (PC1) and prohormone convertase 2 (PC2), leading to the generation of biologically active TRH and other non-TRH peptides with potential biologic activity [10], [20], [21], [28]. We therefore performed RIAs to measure both the amount of TRH and the amount of two other peptides derived from the proTRH precursor: pYE27 from the N-terminus and the C-terminal fragment pYE17.

RIA assays (**Figure 1D**) demonstrated large increases in pYE27 (mixed design ANOVA, Treatment × Side interaction: F_3,15_ = 487.8, p<0.0001), pYE17 (F_3,15_ = 11205, p<0.0001) and TRH (F_3,15_ = 1385, p<0.0001) in the striatum ipsilateral to the 6-OHDA lesion in the animals treated with long-term L-DOPA, but not on the contralateral side, and not in the dopamine-depleted striatum of control rats that were treated with saline, nor in sham animals treated with L-DOPA. These measurements suggested that the TRH that we detected in the striatum of the L-DOPA-treated animals in the dopamine-depleted hemisphere was the result of full processing of proTRH hormone. This finding, in combination with increased *preproTRH* mRNA within the striatum, indicates that the up-regulated TRH is encoded for and synthesized locally within the striatum of rats with dyskinesia-like motor abnormalities.

### Immunohistochemical localization of striatal proTRH peptide

To evaluate the regional distribution of the proTRH and TRH changes observed at the mRNA level and peptide level with qPCR and RIA, we used the rabbit antibody raised against the c-terminal peptide pYE17. In control experiments shown in **Figure S1**, we demonstrated that pre-absorption of anti-pYE17 antibody with the peptide pYE17 (amino acids 241–255, QSPQVEPWDKEPLEE) completely abolished immunostaining, whereas pre-absorption of anti-pYE17 antibody with the n-terminal peptide pYE27 (amino acids 25-50, LPEAAQEEGAVTPDLPGLENVQVRPE) did not affect immunostaining. These findings, indicating the specificity of the anti-pYE17 antibody, confirm the results of Nillni and colleagues [20], [21], [28], [29], [30], [31] and others [32], [33], [34], [35], [36], [37], [38].

**Figure 2:**
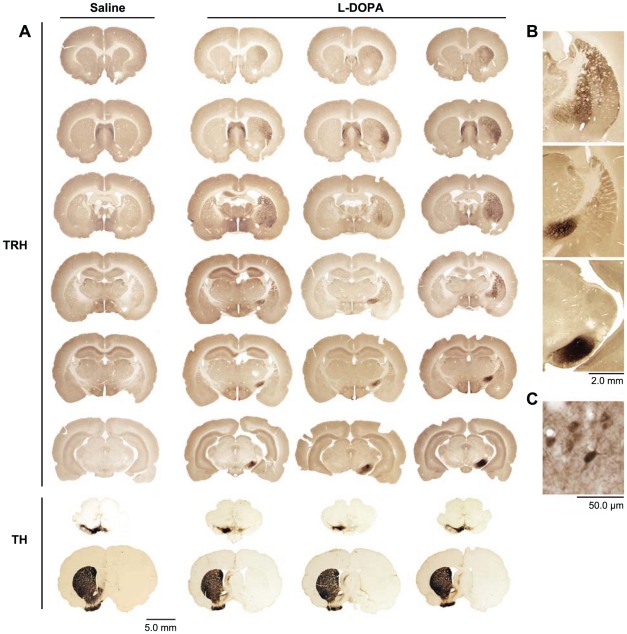
Up-regulation of TRH is specific for the dopamine-depleted striatum of rats with L-DOPA-induced dyskinesias. (**A**) Saline: proTRH immunoreactivity is very low absent in the dopamine-depleted striatum (right) of a control, saline-treated rat. Sections run from rostral (top) to caudal (bottom). L-DOPA: proTRH immunoreactivity is strong and variable in the dopamine-depleted striata of three exemplary L-DOPA-treated dyskinetic rats. Tyrosine hydroxylase (TH) immunostaining indicates loss of dopamine-containing cell bodies in the substantia nigra pars compacta (upper section) and uniform loss of dopamine-containing terminals in the striatum (lower section), ipsilateral to the lesion, of all four rats. (**B**) ProTRH immunostaining in fibers of the external globus pallidus (top panel), entopeduncular nucleus (middle panel) and substantia nigra pars reticularis (bottom panel) of a dopamine-depleted rat with LID. (**C**) ProTRH immunoreactivity in a putative medium spiny neuron in a striatal section from a rat with LID.

With this antibody, we found a striking up-regulation of immunolabeling for proTRH in the dopamine-depleted striatum of the L-DOPA-treated animals, relative to that in control rats treated with saline after the lesion (**Figures 2** **and** **3**).

**Figure 3:**
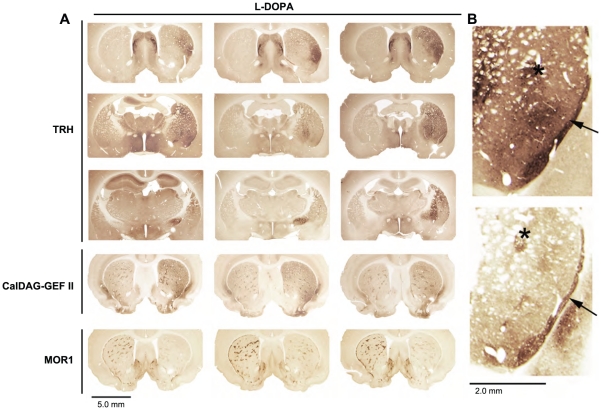
TRH shows a complex pattern of up-regulation that includes preferential expression in caudal striosomes. (**A**) Non-uniform proTRH immunostaining in the dopamine-depleted striatum of three individual rats as compared to relatively uniform up-regulation of CalDAG-GEFII and down-regulation of MOR1 in the same rats. (**B**) The subcallosal streak (arrow) and putative striosome (asterisk) immunostained for proTRH (top panel) and the striosome marker CalDAG-GEFII (bottom panel) in neighboring striatal sections from a rat with LID.

**Figure 4:**
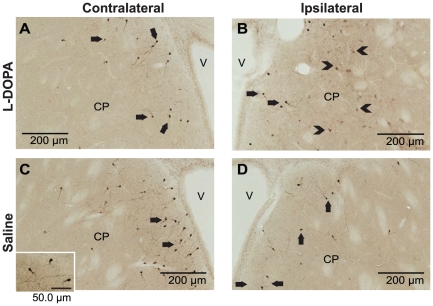
TRH expression in pseudounipolar neurons of rats with LID-inducing treatments and control treatments. (**A**) ProTRH^+^ periventricular neurons in the dorsal striatum **contralateral** to the 6-OHDA lesion in an animal that developed LIDs (magnification 10×). Full arrows show clusters of pseudounipolar neurons. (**B**) *ProTRH* ^+^ periventricular neurons and putative medium spiny neurons in the dorsal striatum **ipsilateral** to the 6-OHDA lesion in an animal that developed LIDs. Full arrows show clusters of pseudounipolar neurons; tail-less arrows show examples of putative medium spiny neurons. (**C**) ProTRH^+^ periventricular neurons in the dorsal striatum **contralateral** to the 6-OHDA lesion in an animal treated with saline (inset: higher magnification of the pseudounipolar neurons immunopositive for proTRH). Full arrows show clusters of pseudounipolar neurons. (**D**) ProTRH^+^ periventricular neurons in the dorsal striatum **ipsilateral** to the 6-OHDA lesion in an animal treated with saline. Full arrows show clusters of pseudounipolar neurons; Abbreviations: striatum: CP; lateral ventricle: V.

In the rats treated with saline (**Figure 2A**), there was almost no proTRH immunostaining visible in the dopamine-depleted striatum, and proTRH immunostaining was also almost absent in the striatum contralateral to the dopamine-depletion in L-DOPA-treated animals. Exceptions were scattered proTRH-positive pseudounipolar neurons in the most dorsal and medial parts of the caudoputamen, near the lateral ventricles (**Figure 4**), present in all animals regardless of their treatment schedule. They resemble neurons of the rostral migratory stream [39], [40].

In sharp contrast, there was strong proTRH immunostaining in the dopamine-depleted striatum of the rats treated with L-DOPA. **Figure 2A** illustrates this gradient of immunostaining at six anteroposterior levels in three of the cases, relative to the immunostaining at similar levels through the brain of a saline-treated control. The L-DOPA-treated cases shown from left to right in **Figure 2A** received, respectively, average dyskinesia scores of 7, 8 and 8 for the last three behavioral assessments. On the dopamine-depleted side, the proTRH immmunostaining was intense laterally and ventrolaterally, but there was relatively little immunostaining medially or at far-anterior levels. Caudally, small focal zones of intense stain appeared, with very little immunostaining around them (**Figure 2B**, top). The L-DOPA treatment also induced intense immunostaining of neuropil in the globus pallidus (the rodent equivalent of the external pallidum), the entopenduncular nucleus (the rodent equivalent of the internal pallidum), and the pars reticularis of the substantia nigra (**Figure 2B**, middle and bottom panels). This proTRH immunostaining of the output nuclei of the striatum was strong in all L-DOPA-treated rats on the side of dopamine depletion (**Figure 2A**, 3 right columns), and was absent in all controls (**Figure 2A**, left column).

The proTRH labeling in the intensely immunostained regions of the caudoputamen was in both cell bodies and neuropil and appeared to be associated with neurons of medium size, likely corresponding to medium spiny neurons (**Figure 2C**). The staining observed in globus pallidus, entopenduncular nucleus and the substantia nigra pars reticularis appeared to be concentrated in neuropil, a pattern consistent with labeling of the axons of striatal medium spiny neurons (**Figure 2B**).

The proTRH immunostaining was not equivalently intense in the dopamine-depleted striatum of all of the animals treated with L-DOPA, but elements of the patterns shown in **Figures 2** **and** **3** appeared in all of them. To test whether this uneven striatal immunostaining was the result of variations in the locations of the 6-OHDA injections or unintended damage during the 6-OHDA injections or fixation process, we stained nearby striatal sections for TH, CalDAG-GEFII and µ opioid receptor 1 (MOR1). TH was chosen as a marker of the extent of the dopamine depletion, whereas CalDAG-GEFII and MOR1 are two immunomarkers that have been previously shown to be dysregulated by combined 6-OHDA lesion and L-DOPA therapy [9], [41].

TH immunostaining of the substantia nigra (**Figure 2A** second to last row) was, as expected, strong on the control side but was scarcely detectable on the side of the 6-OHDA lesion. Similarly, TH immunostaining of the striatum was strong contralateral to the lesion but was almost nil on the dopamine-depleted side. CalDAG-GEFII and MOR1 immunostains also were dysregulated across the full extent of the striatum on the side of the lesion in the L-DOPA-treated animals: CalDAG-GEFII was strongly up-regulated, and MOR1 immunostaining was strongly down-regulated (**Figure 3A**). Thus, the spatial extent of the proTRH up-regulation, favoring the lateral striatum, did not appear to be the result of a partial lesion effect as judged by three other immunomarkers. This observation favors the view that TRH may be up-regulated in only a subset of medium spiny striatal neurons. It is possible that variation in the degree of proTRH immunostaining from case to case reflects temporal variation in the TRH response to L-DOPA. There was clearly strong up-regulation of proTRH immunostaining in both striosomes and matrix in the most intensely stained zones of the caudoputamen, but as the proTRH immunostaining diminished medially, the staining was uneven, and at posterior levels, the small zones of intense immunostaining had the appearance of striosomes. With adjoining sections immunostained for a striosomal marker [42], these focal zones were identified as striosomes (**Figure 3B**). Thus the pattern of TRH regulation in the striatum was one in which proTRH was up-regulated in a large part of the lateral striatum in both striosomes and matrix, but in which there was up-regulation in striosomes of the more weakly affected zones, especially at caudal levels.

## Discussion

Our findings demonstrate that in the classic 6-OHDA model of PD, there is a strong up-regulation of expression of TRH in the dopamine-depleted striatum and its efferent projections to the pallidum and substantia nigra. Further, we show that the enhanced expression of striatal proTRH was associated with the behavioral expression of motor complications in response to long-term L-DOPA therapy. Both *preproTRH* mRNA and TRH peptide levels were increased, and these increases were selective for the combination of 6-OHDA-induced dopamine depletion and L-DOPA treatment. As our RIA findings suggested that the TRH peptide produced was fully processed, the TRH detected was likely biologically active. Remarkably, in the striatum of control animals, whether sham-depleted or dopamine-depleted and treated with saline, immunostaining for proTRH was almost absent. These findings suggest that the up-regulation of TRH was induced by combined dopamine depletion and L-DOPA treatment, not by dopamine depletion alone or by L-DOPA treatment alone. Activation of signaling cascades related to TRH could thus be critically important for understanding the mechanisms giving rise to the occurrence of dyskinesias in parkinsonian conditions.

The model that we employed induced an almost complete lesion of the dopamine-containing neurons in the pars compacta of the substantia nigra, and we administered high doses of L-DOPA. This model thus parallels a relatively advanced stage of PD in which dyskinesias are prevalent, presumably because patients have more pronounced nigral cell loss and are taking increasingly high doses of anti-parkinsonian medication. But the 6-OHDA model does not incorporate a prolonged decline of the population of dopamine-containing neurons in the substantia nigra. Whether TRH up-regulation is specific to severe, late-stage models of the disease process or might be observed with lesser degrees of dopamine depletion is still unknown.

We found a marked increase in the mRNA for *preproTRH*, which gives rise to TRH and several other fragments. By immunohistochemistry, we observed a pronounced and patterned increase in proTRH immunostaining in the striatum, with the strongest proTRH expression concentrated in the lateral and ventrolateral striatum including the sensorimotor striatum. This proTRH immunostaining was apparent in the cell bodies of putative striatal medium spiny neurons, and in striatal efferent axons terminating in the external and internal pallidal segments and the pars reticularis of the substantia nigra. Our findings thus suggest that the striatal up-regulation of TRH affects both the direct and indirect pathways of the basal ganglia and parts of the striosomal pathway as well.

We could identify several features of this pronounced increase in striatal TRH. First, the proTRH up-regulation within the striatum mainly occurred in lateral and ventrolateral regions, despite the fact that the dopamine-depletion was relatively uniform. This selectivity suggests that the TRH dysregulation strongly affected the sensorimotor striatum and nearby regions. In addition, there appeared to be differential up-regulation of TRH expression in caudal parts of the striosomal system, judging from the immunohistochemistry. This anatomical distribution accords with the induction of the motor problems in the animals, and is consonant with reports that up-regulation of prodynorphin and FosB is non-uniform and particularly concentrated in parts of the striosomal system and the ventrolateral aspect of the striatum in other rat models of LIDs [8], [43], [44], [45]. The particular emphasis on the lateral and caudal striatum is interesting, given the posteriolateral to anterior gradient of dopamine loss in the human striatum in PD [46].

Second, although we could not conclusively identify the subtype of the medium-sized neurons expressing increased proTRH immunoreactivity, the increased proTRH immunostaining in both pallidal segments and the substantia nigra suggests that both direct and indirect pathway neurons of the striatum were affected by the L-DOPA treatment in such a way that proTRH-immunostained material reached the terminals of these pathways. This finding is interesting in light of findings that L-DOPA-induced hyper-activation of the extracellular signal-regulated kinases, ERK1/2, which are required for the development of LIDs in mouse models, is restricted to medium spiny neurons of the direct pathway [47], [48].

Immunohistochemical stains identified striosomal proTRH up-regulation in striosomes of the caudal striatum. This result is particularly striking, as it suggests that the TRH up-regulation induced by L-DOPA in the dopamine-depleted striatum differentially affects subcircuits within the direct, indirect and striosomal pathways. These subcircuits could be particularly relevant to the genesis of dyskinesias [3], [7], [8], [49], [50] as well as dysfunction in a range of other movement disorders [51], [52], [53]. Notably, the increased striatal TRH immunostaining that we observed was combined with increased immunostaining for CalDAG-GEFII but with decreased immunostaining for MOR1, both reliable markers of striosomes. Interactions between the TRH changes we observed and other known regulatory changes in striatal neurotransmission are likely to be important [5], [54], [55], [56].

Our findings add to a body of evidence that TRH, although best known for its hypophysiotropic function in regulating TSH release and thyroid function, also directly affects non-hypothalamic neural circuits. Hyperthyroidism has long been associated with hyperkinetic movement disorders, and treating this hormonal abnormality can resolve the hyperkinesia [11], [12], [13], [14]. Thyrotoxicosis has been reported in some PD patients with severe tremor [15], [16], [17], [18], and anti-thyroid treatment has been effective in controlling ‘on-off’ phenomenon and dyskinesia in thyrotoxic patients with PD [16], [19]. Our findings raise the possibility that the dyskinesias suffered by patients with PD following prolonged L-DOPA therapy are also correlated with spatially selective dysregulation of TRH within the striatum. Striatal changes in TRH peptide content are associated with changes in mRNA for *preproTRH*, and we found full processing of the prepro-peptide within this same structure. Thus, as the encoding and synthesis of the peptide happens fully within the striatum, it seems likely that the effects of TRH in response to LIDs did not rely on the regulation of peripheral thyroid hormones, which would require release of TRH into the portal system.

Particularly relevant to the findings we report here are experiments suggesting that the behavioral patterns stimulated by increased intrastriatal TRH in intact non-primate mammals are akin to human dyskinesia [57], [58], [59], [60], [61], [62], [63]. Studies have suggested that the behavioral effects of TRH are exerted via the stimulation of striatal dopamine release [64], and that they can be antagonized by both D1-class and D2-class dopamine receptor antagonists [57], [58], [59]. In turn, striatal dopamine levels have also been shown to modulate the release of TRH within the rat striatum [61], [62], with higher levels of dopamine correlating with higher levels of intrastriatal TRH.

Our findings suggest that up-regulation of striatal expression of TRH in the striatum and its output pathways is strongly correlated with the expression of L-DOPA induced dyskinesia and that this up-regulation could be an essential feature of the dyskinetic state in parkinsonism. These observations raise the possibility that treatments targeting TRH expression, the receptor for this biologically active peptide, or related signaling molecules, could be useful in treating or preventing L-DOPA-induced dyskinesias in human PD.

## Materials and Methods

### Subjects and Surgery

Male Sprague-Dawley rats (250–350 g) were anesthetized with a combination of ketamine hydrochloride (75 mg/kg) and xylazine (10 mg/kg). Under stereotaxic guidance, 6 µl of 6-OHDA hydrobromide (10 mM 6-OHDA in 0.01% ascorbic acid, 1 µl/min) or saline (for control, sham-lesion animals) was injected into the right medial forebrain bundle (AP = −4.0 mm, ML = −1.3 mm, DV = 8.4 mm). Three weeks after the surgery, an observer blind to the treatment condition counted the number of turns made to the side contralateral to the side of the 6-OHDA lesion over a 1 min sampling period, 10 min after injection of apomorphine subcutaneously (0.5 mg/k). This procedure was followed to estimate the extent of dopamine depletion. Rats with 6 or more contralateral turns/min were classified as having a successful lesion.

### Drug Treatments and Behavioral Observation

The rats were treated intraperitoneally (i.p.) with either L-DOPA (25 mg/kg)/benserazide hydrochloride (6.25 mg/kg) in 1 ml/kg saline solution, or with saline (1 ml/kg), for a period of 21 days twice daily (10 AM and 5 PM). **Table 1** shows the numbers of rats and the test group assignment of all of the animals.

**Table 1:** Experimental groups.

Test	Lesion and Drug Treatment	N
qPCR	Lesion + Saline	11
	Lesion + L-DOPA	10
	Sham lesion + Saline	2
	Sham lesion + L-DOPA	3
RIA; IHC	Lesion + Saline*	8
	Lesion + L-DOPA **	9
	Sham lesion + Saline***	2
	Sham lesion + L-DOPA****	3

Description of the assignment of the animals used in this study to the different experimental groups, and the biological tests they were used for (

*4 Fixed and 5 Frozen;

**4 Fixed and 5 Frozen;

***1 Fixed and 1 Frozen;

****1 Fixed and 2 Frozen).

Abbreviations: IHC, immunohistochemistry.

The rats were observed for 1 min, 20 min after the administration of L-DOPA on every third day during the entire treatment period. Turning behavior contralateral to the lesion side was recorded as a measure of the severity of the lesions. Dyskinetic behaviors were recorded and subsequently coded on the basis of the type and severity of the behaviors exhibited (**Table 2**) by two observers blind to the treatment. The animals were euthanized 12 hrs after the last injection (see **Table 1**). All of the animal treatments were approved by the Massachusetts Institute of Technology (MIT) Committee on Animal Care.

**Table 2:** Behavioral rating scale for L-DOPA-induced dyskinesias.

Abnormal Involuntary Movements	Severity	Rating
Contralateral Involuntary Repetitive Forelimb Movements	Absent:	0
	Mild:	1
	Moderate:	2
	Severe:	3
Twisted Posture of Head and/or Body to Contralateral Side	Absent:	0
	Mild:	1
	Moderate:	2
	Severe:	3
Contralateral Turning Behavior	≤6/minute:	1
	≥6/minute:	2
Hindlimb Dystonia	Absent:	0
	Present:	1
Oral Stereotypy	Absent:	0
	Present:	1

### RNA Extraction and Real-Time qPCR

Rats were decapitated, brains were rapidly removed from the calvarium, and striata were rapidly dissected out and frozen. Total RNA was isolated from the dissected striatal samples and cDNA was synthesized as previously described [27]. Short synthetic PCR primers were designed to amplify a small 225 bp amplicon for *preproTRH* (forward primer: TGT CAC CAA GAG GCA ACA TC; reverse primer: CTT TGC TTC ACC AGG GTC TC; annealing temperature 60°C) using Primer3 software (http://frodo.wi.mit.edu/primer3/). qPCR was carried out using an iCycler® (BioRad, Hercules, CA), and SYBR® Green PCR Master Mix (Applied Biosystems, Foster City, CA). Curves with known concentrations of cDNA were generated to calculate the efficiency of each of the primers, and to quantitate products with the ΔΔCt method [65], [66]. Data were normalized to *GAPDH* mRNA levels.

### Peptide Radioimmunoassay (RIA)

All RIAs were performed as previously described [20]. The RIA incubation contained 100 pl of antiserum, 100 pl of sample, and 200 pl of RIA buffer (10.1 M phosphate buffer with 0.5% bovine serum albumin; pH 7.2). One hundred µl of ^125^I-peptide (10,000 cpm) was added following a 24 hr pre-incubation at 4°C. After an additional 48 hr incubation at 4°C, 1.0 ml of 0.1% activated charcoal was added to each reaction to separate bound and free ^125^I-peptide. The supernatants were counted for radioactivity, following centrifugation at 2,000× g for 30 min.

### Immunohistochemistry

Rabbit anti-CalDAG-GEFII (1∶1,500, Santa Cruz Biotechnology); mouse anti-tyrosine hydroxylase (1∶4,000, Immunostar); rabbit anti-pYE17 (1∶4000; precursor of TRH was generated in the laboratory of Eduardo Nillni [20]), and rabbit anti-µ opioid receptor 1 (1∶20,000, Immunostar) were used for immunohistochemistry for light microscopy as described in [67].

### Statistical Methods

#### Behavioral testing

The dyskinesia scores were analyzed with Friedman's two-way ANOVA by ranks for correlated samples, followed by Dunn's test for multiple comparisons. To estimate the degree of correlation between mRNA levels and behavioral measures, we calculated Spearman's correlation coefficients for ranked data.

#### qPCR and RIA measurements

A mixed design ANOVA with a between subjects variable (Treatment: saline, L-DOPA), and a within subject variable (Side: lesion, no lesion) was used to evaluate statistical differences between left and right striatal mRNA levels and RIA peptide levels for samples from animals with 6-OHDA lesions treated with chronic L-DOPA or saline, followed by a Bonferroni T test for multiple comparisons.

## Supporting Information

Figure S1 Pre-absorption of anti pYE17 antibody demonstrates the specificity of proTRH immunostaining. For this experiment we used a rat with a 6-OHDA lesion and long-term levodopa treatment. This animal developed LIDs and upregulation of TRH. We immunostained striatal sections from this rat with anti-pYE17 alone, anti-pYE17 pre-absorbed with either pYE17 or pYE27 peptide or with the primary antibody omitted. (A) Anti-pYE17 alone shows strong immunostaining for proTRH. (B) Immunostaining was completely blocked by preabsorbtion of anti-pYE17 with pYE17 peptide. (C) Immunostaining for proTRH was not disrupted by preabsorbtion of anti-pYE17 with pYE27 peptide. (D) Omission of the primary antibody anti-pYE17 abolished immunostaining.
(0.35 MB EPS)
